# Characterization of Solitary Lesions in the Extremities on Whole-Body Bone Scan in Patients With Known Cancer: Contribution of Single-Photon Emission Computed Tomography/Computed Tomography

**DOI:** 10.3389/fonc.2019.00607

**Published:** 2019-07-09

**Authors:** Hao Peng, Linqi Zhang, Tao Zhou, Wei Li, Wen Li, Liwu Ma, Rusen Zhang

**Affiliations:** Department of Nuclear Medicine, Affiliated Cancer Hospital & Institute of Guangzhou Medical University, Guangzhou, China

**Keywords:** solitary lesion, extremities, ^99m^Tc-MDP, SPECT/CT, whole-body bone scan

## Abstract

**Background:** Solitary lesions in the extremities showing ^99m^Tc-methylene diphosphate (MDP) uptake are often encountered on whole-body bone scan (WBS), and proper interpretation of this diagnostic method is important for patients with known cancer. The purpose of this study was to summarize the characteristics of solitary lesions in the extremities of patients with known cancer and to evaluate the diagnostic accuracy of single-photon emission computed tomography/computed tomography (SPECT/CT) in differentiating bone metastases from benign bone lesions.

**Methods:** This study was a retrospective review of 86 patients (54 males and 32 females; mean age, 57.88 ± 10.97 years; range, 31–81 years) with known cancer who underwent WBS and showed solitary lesions with ^99m^Tc-MDP uptake in the extremities and then underwent SPECT/CT for further diagnosis. SPECT/CT images were independently interpreted by two experienced nuclear medicine physicians. The diagnostic accuracy of SPECT/CT in differentiating malignant from benign solitary lesions in the extremities was evaluated. Inter-reviewer agreement was assessed by using weighted *k* statistics. The standard diagnostic criterion was based on biopsy or radiologic follow-up over at least 12 months.

**Results:** In total, 23 bone metastases and 63 (73.26%) benign lesions were diagnosed. The majority (16/23, 69.57%) of bone metastases were found in the diaphyses. The most common benign bone disease was a benign bone tumor (31.75%, 20/63). The majority (13/20, 65%) of benign bone tumors were enchondromas. In the proximal and distal extremities, the most common disease was degeneration (27.11%, 16/59), followed by benign bone tumors and osteonecrosis of the femoral head (ONFH) (22.03%, 13/59). In the diaphyses of the extremities, bone metastasis was the most common disease, accounting for 64% (16/25) of the findings. For the SPECT/CT analysis, the accuracy was 94.19% (81/86) for reviewer 1 and 95.34% (82/86) for reviewer 2. The weighted kappa score for inter-reviewer agreement was 0.813.

**Conclusion:** When solitary disease of the extremities is detected by WBS in patients with known cancer, benign lesions may be more common than malignant lesions. SPECT/CT resulted in not only fewer equivocal lesions but also in higher diagnostic accuracy.

## Introduction

^99m^Tc-labeled methylene diphosphonate (^99m^Tc-MDP) whole-body bone scan (WBS) is commonly used for detecting bone metastases in patients with known cancer with high sensitivity but low specificity (1). The extremities are not the most common site of bone metastasis, and only 9% of bone metastases initially appear as a single focus on WBS (2). However, some investigators have reported that some benign bone diseases that showed significantly increased ^99m^Tc-MDP uptake in the extremities on WBS may mimic bone metastasis and should be further distinguished, especially in patients with known cancer (3–5). Currently, single-photon emission computed tomography/computed tomography (SPECT/CT) is superior to WBS alone in differentiating benign from malignant lesions (6–8). SPECT/CT not only provides information about the location, matrix changes, and appearance of soft tissues around the bone lesion but also characterizes abnormal radiotracer uptake (7, 9). To our knowledge, many studies have revealed that SPECT/CT increases the diagnostic accuracy for lesions identified on WBS (6, 7, 9, 10). However, the diagnostic accuracy of SPECT/CT in differentiating malignant from benign solitary lesions in the extremities detected by WBS is still unclear (3, 6, 7, 9). The literature on the general characteristics of solitary lesions in the extremities is sparse (3). Accordingly, the aim of our study was to summarize the characteristics of solitary lesions with ^99m^Tc-MDP uptake in the extremities on WBS in patients with known cancer and to evaluate the diagnostic accuracy of SPECT/CT in differentiating bone metastasis from benign bone lesions.

## Methods

### Patients

Between March 2009 and March 2016, we undertook a single-center retrospective diagnostic study. The criteria for inclusion in the study were as follows: (1) patients with known malignancy, (2) patients who underwent ^99m^Tc- MDP WBS for screening, (3) patients with areas of the extremities with abnormal radiotracer uptake who underwent SPECT/CT for further diagnosis, and (4) patients with more than 12 months of follow-up imaging after undergoing SPECT/CT. In addition, we excluded diseases with a definitive diagnosis of bone metastasis on WBS and without a definitive diagnosis within the follow-up period. Patients with benign bone lesions (such as fracture and degeneration) in the axial skeleton were not excluded. This study was approved by the local ethics committee, and because of the retrospective nature of the study, written informed consent was omitted.

### Image Acquisition

All acquisitions were performed using a Philips SPECT/CT scanner (Netherlands). WBS was carried out 2–3 h after the intravenous injection of ^99m^Tc-MDP at a dose of 15–25 mCi. After WBS acquisition, the images were directly interpreted by an experienced nuclear medicine physician. If doubtful lesions were identified on WBS, the addition of SPECT/CT was then immediately performed for anatomical localization and further diagnosis. First, a low-dose CT was performed for anatomic location and attenuation correction. CT data were acquired with exposure of 120 KV, 100 mAs/slice, window width of 15%, pitch of 1.25, and slice thickness of 5.0 mm. After CT acquisition, the SPECT acquisition protocol was started. The acquisition parameters for SPECT were as follows: 15% energy window at 140 keV and 64 × 64 matrix. Processing of the WBS and SPECT/CT images was performed on Jet Steam Workspace (Philips).

### Data/Image Analysis

Images were independently interpreted by two experienced nuclear medicine physicians with knowledge of the history of malignancy. On WBS analysis, all 86 lesions were interpreted as equivocal. All bone lesions were classified on a 3-point scale as benign, malignant or equivocal on SPECT/CT. Malignant lesions were suggested by the presence of lytic, sclerotic, or mixed changes and a soft tissue mass. Furthermore, the presence of osteophytes or subchondral sclerosis was regarded as a clear sign of benignity. Lesions were classified as equivocal if they could not be confidently diagnosed as malignant or benign.

We evaluated the following features of the lesion: (1) location and (2) degree of osteoblastic activity. High activity was considered if the lesion showed uptake of ^99m^Tc-MDP higher than that of the sternum on WBS images, moderate activity was considered if the lesion uptake was equal to that of the sternum, and low activity was considered if the lesion uptake was lower than that of the sternum.

### Pathology and Follow-Up

The final decision regarding the true status of lesions was based on biopsy or follow-up imaging, which was made after consideration of all the available clinical information, including histological confirmation. This was then used as a criterion standard for this study. Pathologic analysis confirmed the diagnosis in 13 cases. In the remaining 73 cases, the patients had been diagnosed based on radiologic investigations (WBS and/or SPECT/CT, MRI) and follow-up of at least 12 months.

### Statistical Analysis

Categorical data are expressed as numbers and frequencies (%). Continuous data are expressed as the means and standard deviations. Intergroup differences in the location and osteoblastic activity were analyzed using chi-square tests. The results were considered significant for a *P*-value of <0.05.

To evaluate the diagnostic accuracy of SPECT/CT for solitary lesions in the extremities, the sensitivity, specificity, and accuracy were calculated. Inter-reviewer agreement was estimated using a weighted *k*-statistic. A 95% confidence interval (CI) was chosen to determine the significance between groups, with *P*-values <0.05 indicating significant differences. The *k*-statistic was used for inter-reviewer reliability analysis. The *k*-values were interpreted as indicating poor (*k* < 0), slight (0 < *k* < 0.2), fair (0.21 < *k* < 0.4), moderate (0.41 < *k* < 0.6), substantial (0.61 < *k* < 0.8), and almost perfect (0.81 < *k* < 1.0) inter-observer agreement (11, 12). All statistical tests were performed using SPSS Statistics 17.0 software (SPSS Inc., Chicago, IL, USA).

## Results

### Patient Population

A total of 86 patients with ^99m^Tc-MDP uptake in the extremities on WBS were included. Fifty-four patients were men, and 32 were women, ranging in age from 31 to 81 years (mean age 57.88 ± 10.97 years). The primary malignancies included lung cancer (*n* = 30), nasopharyngeal cancer (*n* = 19), breast cancer (*n* = 12), cervical cancer (*n* = 5), rectal cancer (*n* = 4), esophageal cancer (*n* = 4), hepatic cancer (*n* = 3), prostatic cancer (*n* = 3), and colon cancer (*n* = 2), and one patient each had hypopharyngeal cancer, laryngeal cancer, gastric cancer, endometrial cancer, as summarized in Table 1.

**Table 1:** Type and incidence of malignant disease and the relation with extremity lesions.

**Type of malignancy**	**Value**	**Extremity lesions**		**Percentage**
		**Metastasis**	**Benign**	
Lung cancer	30	11	19	34.88%
Nasopharynx cancer	19	6	13	22.09%
Breast cancer	12	2	10	13.95%
Cervical cancer	5	0	5	5.81%
Rectal cancer	4	2	2	4.65%
Esophageal cancer	4	0	4	4.65%
Hepatic cancer	3	2	1	3.49%
Prostatic cancer	3	0	3	3.49%
Colon cancer	2	0	2	2.33%
Hypopharyngeal cancer	1	0	1	1.16%
Laryngeal cancer	1	0	1	1.16%
Gastric cancer	1	0	1	1.16%
Endometrial cancer	1	0	1	1.16%

### Patient Analysis

On the basis of the criterion standard, 63 (73.26%) lesions were diagnosed as benign bone disease, whereas 23 (26.74%) were determined to be malignant. All 86 lesions were classified into six types, as detailed in Table 2.

**Table 2:** Bone disease types among 86 patients.

	**Number of patients**	**Percentage**
Metastasis	23	26.74%
Benign bone tumor	20	23.26%
Enchondroma	13	65%^b^
Osteochondroma	2	10%^b^
Osteoid osteoma	1	5%^b^
Enostosis	1	5%^b^
FD	2	10%^b^
NOF	1	5%^b^
Fracture	12	13.95%
Degeneration	16	18.60%
ONFH	13	15.12%
Other*^*a*^*	2	2.32%

FD, fibrous dysplasia; NOF, nonossifying fibroma; ONFH, osteonecrosis of the femoral head.

a Other included chronic osteomyelitis and Paget's disease of bone.

b *The Percentage of benign bone tumors in 20 cases*.

### Site and Intensity of ^99m^ Tc-MDP Uptake

**Figure 1:**
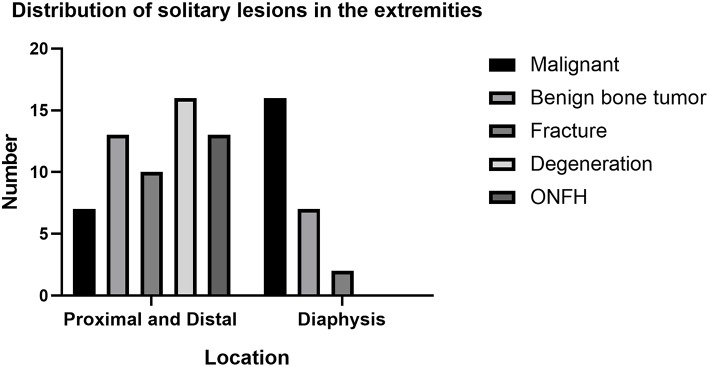
Distribution of solitary lesions in the extremities.

**Figure 2:**
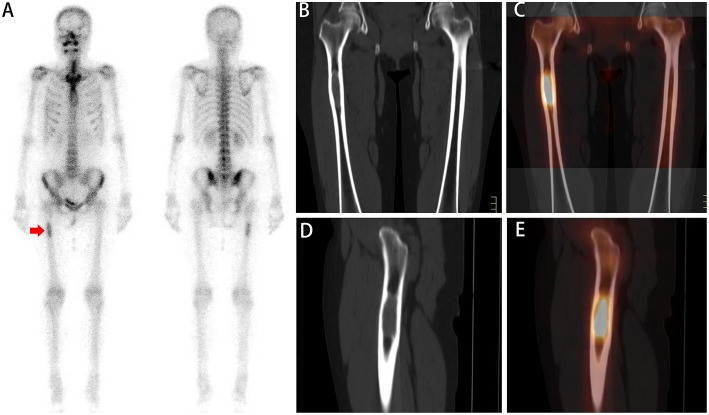
A whole-body scan **(A)** revealed a focal area of low ^99m^Tc-MDP activity in the right femur (red arrow). No additional areas of abnormal ^99m^Tc-MDP uptake were identified in the remainder of the skeleton. Coronal **(B,C)** and sagittal **(D,E)** CT and SPECT/CT imaging showed focally increasing ^99m^Tc-MDP uptake corresponding to lytic changes and soft tissue masses in the diaphysis of the femur. A biopsy was subsequently performed, and pathologic analysis confirmed the diagnosis of bone metastasis.

Two lesions (diagnosed as chronic osteomyelitis and Paget's disease of bone) showed diffuse areas of radiotracer uptake in a single bone, while the remaining 84 lesions showed focal radiotracer uptake on WBS. The anatomical location and final diagnosis of these 84 lesions are shown in Table 3. In proximal and distal extremities, benign bone disease was more frequently identified than malignant disease (Figure 1). The most common disease was degeneration (27.11%, 16/59), followed by benign bone tumors and ONFH (22.03%, 13/59). In the diaphyses of the extremities, bone metastasis was the most common disease, accounting for 64% (16/25) of the findings (Figure 2). When excluding disease with a diffuse area of radiotracer uptake on WBS, the difference in location between two groups was significant (χ^2^ = 24.00, *P* < 0.01) (Table 4).

**Table 3:** Anatomical location and final diagnosis of 84 extremity lesions.

		**Metastasis**	**Benign**
Humerus	diaphysis/proximal/distal	3/3/0	3/14/0
Ulna	diaphysis/proximal/distal	0/0/0	0/2/1
Femur	diaphysis/proximal/distal	12/4/0	3/29/1
Tibia	diaphysis/proximal/distal	1/0/0	3/0/5
**Top three tumor locations**			
Proximal part of femur (%)		4 (4.76%)	29 (34.52%)
Proximal part of humerus (%)		3 (3.57%)	14 (16.67%)
Diaphysis part of femur (%)		12 (14.28%)	3 (3.57%)

**Table 4:** The relation between localization/degree of osteoblastic activity and final diagnosis of extremity lesions.

	**Localization**		**Degree of osteoblastic activity**	
	**Proximal/distal**	**Diaphysis**	**High**	**Moderate/low**
Malignant	7	16	13	10
Benign	52	9	40	21
*P*-value	<0.001		0.443	

The intensity of ^99m^Tc-MDP uptake in malignant bone disease was considered high in 13 lesions and moderate or low in 10 sites. The intensity of uptake in benign diseases was considered high in 40 sites and moderate or low in 21 sites. The osteoblastic activity was not significantly different between the two groups (χ^2^ = 0.588, *P* > 0.05) (Table 4).

### SPECT/CT Diagnostic Efficacy

**Figure 3:**
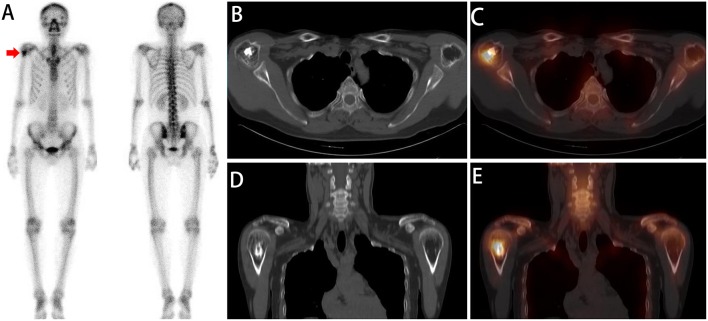
A whole-body scan **(A)** revealed a focal area of high ^99m^Tc-MDP activity in the right humerus (red arrow). No additional areas of abnormal ^99m^Tc-MDP uptake were identified in the remainder of the skeleton. Axial **(B,C)** and coronal **(D,E)** CT and SPECT/CT imaging showed focally increased ^99m^Tc-MDP uptake corresponding to ossification in the proximal part of the humerus. The diagnosis of enchondromas was established by a combined assessment of clinical and radiologic follow-up.

**Figure 4:**
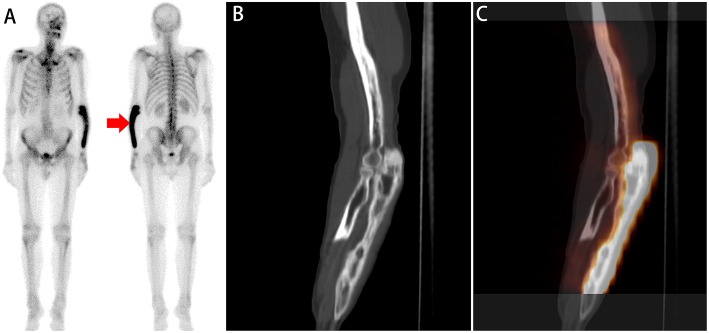
A whole-body scan **(A)** revealed diffuse high ^99m^Tc-MDP activity in the left forearm (red arrow). No additional areas of abnormal ^99m^Tc-MDP uptake were identified in the remainder of the skeleton. Sagittal **(B,C)** CT and SPECT/ CT imaging showed diffusely increased ^99m^Tc-MDP uptake corresponding to irregular cortical thickening and porous sclerosis in the ulna. The diagnosis of Paget's disease of bone was established by a combined assessment of clinical and radiologic follow-up.

**Figure 5:**
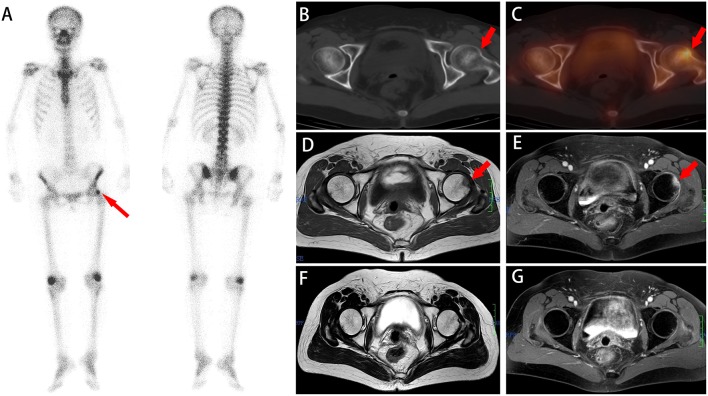
A whole-body scan **(A)** revealed a focal area of high ^99m^Tc-MDP activity in the left femur (red arrow). No additional areas of abnormal ^99m^Tc-MDP uptake were identified in the remainder of the skeleton. Axial **(B,C)** CT and SPECT/ CT imaging showed focally increased ^99m^Tc-MDP uptake without density changes in the proximal part of the femur. Axial **(D,E)** MR images show high signal on T1WI and T2WI that disappeared 5 months later **(F,G)**. The diagnosis of IF was established based on a combined assessment of clinical and radiologic follow-up.

All 86 lesions detected on WBS were interpreted as equivocal. On SPECT/CT analysis, for reviewer 1, 64 lesions with benign findings were interpreted as benign (Figures 3, 4), and 19 lesions with malignant findings were interpreted as bone metastases. Only three lesions were interpreted as equivocal on SPECT/CT. Based on the standard criterion, one of three equivocal lesions were diagnosed as benign (Figure 5), and two lesions were diagnosed as malignant. Reviewer 2 reclassified 61 lesions with benign findings as benign and 20 lesions with malignant findings as bone metastases. Only five lesions were interpreted as equivocal. Based on the standard criterion, three of five lesions were diagnosed as benign (Figure 5), and two lesions were diagnosed as malignant. Considering equivocal lesions as positive, the accuracy, specificity, and sensitivity for reviewer 1 were 94.19% (81/86), 86.96% (20/23), and 96.82% (61/63), respectively. The accuracy, specificity, and sensitivity for reviewer 2 were 95.34% (82/86), 95.65% (22/23), and 95.24% (60/63), respectively (Table 5). The inter-reviewer agreements were almost perfect. The k score for inter-reviewer agreement was 0.813 (*P* < 0.001, 95% CI 0.661–0.964) for SPECT/CT.

**Table 5:** Diagnostic performance of SPECT/CT in 86 extremity lesions.

	**Accuracy**	**Specificity**	**Sensitivity**
Reviewer 1	94.19% (81/86)	86.96% (20/23)	96.82% (61/63)
Reviewer 2	95.34% (82/86)	95.65% (22/23)	95.24% (60/63)

*CT, computed tomography; SPECT, single-photon emission computed tomography*.

## Discussion

Final diagnoses of the 86 solitary lesions detected on WBS showed that 73.26% were benign bone lesions and 26.74% were bone metastases. As a rare disease, the incidence of solitary bone metastases accounts for only 2–3% of all skeletal spread situations (13). Compared to the extremities, vertebrae are the most common sites of bone metastases (13). Furthermore, recent studies have shown that the number of solitary bone metastases is less than the number of benign lesions in the spine (10, 14). Considering that only 10% of bone metastases occur in the limbs, which is a lower rate than that in the spine (15), the incidence of benign disease is obviously higher than that of bone metastases. The study performed by Daisuke Utsunomiya (6), which had similarities to this study, recruited 45 patients with a total of 82 areas of abnormal tracer uptake on WBS and found the majority of metastases located in the axial skeleton (83.33%, 35/42) and only four in the femur. L. C. McLoughlin recruited 54 male prostate cancer patients into the study, and only three of 13 bone metastatic lesions were located in the extremities (8). Therefore, we assumed that benign bone disease could be even more common than bone metastases of solitary lesions in the extremities.

In our study, we found that the most common begin bone disease was benign bone tumors (31.75%, 20/63). To our knowledge, degeneration is a common pathology among the elderly, and approximately half of the lesions in our groups belonged to patients who were older than 60 years. However, the number of benign bone tumors is similar to the number of lesions that were comorbid with degeneration in our groups, which may indicate that not all cases of degeneration show obvious radiotracer uptake on WBS or SPECT/CT. Therefore, we assumed that the actual incidence of degeneration may be higher than that of benign bone tumors. In addition, the majority (13/20.65%) of benign bone tumors were enchondromas, which were frequently found in the proximal femur, accounting for 61.53% (8/13). Although enchondroma occurs mostly in the metaphysis (16, 17), only 2.6% of all benign bone tumors are enchondromas (18–25). On the one hand, the majority of enchondromas are asymptomatic and most often identified incidentally for other reasons (26–28). Hence, we assumed that the incidence of enchondromas may be higher than that reported. On the other hand, with their distinct mineralization, most enchondromas show high tracer uptake on WBS (29). In our trial, all enchondromas showed high activity on WBS, and we assumed that enchondroma is easy to detect on WBS.

The locations of 84 focal lesions in the extremities were documented and evaluated to determine the correlation with diagnosis. In the proximal and distal extremities, the most common disease was degeneration (27.11%, 16/59), followed by benign bone tumors and ONFH (22.03%, 13/59). In the diaphyses of the extremities, bone metastasis was the most common disease, accounting for 61.53% (8/13). This may suggest that degeneration and ONFH are not only common among the elderly but also frequently occur in the proximal or distal extremities near the joints. In addition, many benign bone tumors (such as enchondroma and osteochondroma) occur mostly in the metaphysis (16). The findings of Rikard W. also revealed that metastases located in the humeral diaphysis were the most common, followed by proximal and distal lesions (30). Accordingly, it was important that solitary lesions in the proximal or distal parts of the extremities were usually benign, and diaphyseal lesions were usually malignant. The osteoblastic activity was not significantly different between metastases and benign lesions.

However, provided only limited spatial resolution and anatomical correlation, WBS may often be difficult to differentiate between bone metastasis and a benign lesion (31, 32). SPECT/CT not only precisely matched images of body structure and degree of osteoblastic activity in one imaging session but also enhanced the specificity of characteristics of lesions on CT, which assisted in distinguishing benign from malignant lesions (9, 31). In our study, we found that SPECT/CT exhibited a high sensitivity and specificity in differentiating malignant from benign solitary lesions in the extremities and showed almost perfect inter-reviewer agreement. Previous work in other patient cohorts supports the high specificity of SPECT/CT (7, 33, 34).

Our study had several limitations. First, a histopathological diagnosis was not available for all patients, especially for those with degenerative or fracture lesions. The final diagnosis of 73 lesions was based on radiological investigations (WBS and/or SPECT/CT, MRI), clinical information, and follow-up imaging. Second, some solitary lesions without radiotracer uptake (such as osteolytic bone metastases) could have been missed. Third, the number of patients included in the study was relatively small.

In summary, as for the interpretation of solitary bone disease in the extremities of patients with known cancer detected by WBS, SPECT/CT showed great diagnostic efficiency and was significant in differentiating malignant from benign solitary lesions in the extremities. Benign bone disease (such as benign bone tumors and degeneration) may be even more common than malignancy among solitary lesions in the extremities. Bone metastases were found more frequently in the diaphyses of the extremities rather than in other bone areas. Degeneration was the most frequent disease located in the proximal extremities, followed by benign bone tumors and ONFH.

## Data Availability

The datasets generated for this study are available on request to the corresponding author.

## Ethics Statement

The current study was approved by the Institutional Ethics Committee of the Affiliated Cancer Hospital & Institute of Guangzhou Medical University (No. 2017003), and the need for signed informed consent was waived.

## Author Contributions

HP and LZ participated in the design of the study and drafted the manuscript and TZ collected the patients' data. WeiL and WenL interpreted the WBS and SPECT/CT images and LM processed the figures. RZ conceived the study and supervised the project. All authors read and approved the final version of the manuscript.

### Conflict of Interest Statement

The authors declare that the research was conducted in the absence of any commercial or financial relationships that could be construed as a potential conflict of interest.

## Abbreviations

^99m^Tc-MDP: ^99m^Tc-labeled methylene diphosphonate
WBS: whole-body bone scans
SPECT/CT: single-photon emission computed tomography/computed tomography.
